# Advanced glycation end products increase lipids accumulation in macrophages through upregulation of receptor of advanced glycation end products: increasing uptake, esterification and decreasing efflux of cholesterol

**DOI:** 10.1186/s12944-016-0334-0

**Published:** 2016-09-19

**Authors:** Lei Xu, Yi-Ru Wang, Pei-Cheng Li, Bo Feng

**Affiliations:** 1 Department of Endocriology and Metabolic Disease, East Hospital, Tongji University School of Medicine, Shanghai, 200120 China; 2 Tongji University School of Medicine, Shanghai, 200120 China; 3 Ji-mo Road 150, Shanghai, 200120 China

**Keywords:** AGEs, RAGE, Macrophages, Diabetes, Atherosclerosis, Cholesterol

## Abstract

**Background:**

Previous reports have suggested that advanced glycation end products (AGEs) participate in the pathogenesis of diabetic macroangiopathy. Our previous study have found that AGEs can increase the lipid droplets accumulation in aortas of diabetic rats, but the current understanding of the mechanisms remains incomplete by which AGEs affect lipids accumulation in macrophages and accelerate atherosclerosis. In this study, we investigated the role of AGEs on lipids accumulation in macrophages and the possible molecular mechanisms including cholesterol influx, esterification and efflux of macrophages.

**Methods:**

THP-1 cells were incubated with PMA to differentiate to be macrophages which were treated with AGEs in the concentration of 300 μg/ml and 600 μg/ml with or without anti-RAGE (receptor for AGEs) antibody and then stimulated by oxidized-LDL (oxLDL) or Dil-oxLDL. Lipids accumulation was examined by oil red staining. The cholesterol uptake, esterification and efflux were detected respectively by fluorescence microscope, enzymatic assay kit and fluorescence microplate. Quantitative RT-PCR and Western blot were used to measure expression of the moleculars involved in cholesterol uptake, synthesis/esterification and efflux.

**Results:**

AGEs increased lipids accumulation in macrophages in a concentration-dependent manner. 600 μg/ml AGEs obviously upregulated oxLDL uptake, increased levels of cholesterol ester in macrophages, and decreased the HDL-mediated cholesterol efflux by regulating the main molecular expression including CD36, Scavenger receptors (SR) A2, HMG-CoA reductase (HMGCR), ACAT1 and ATP-binding cassette transporter G1 (ABCG1). The changes above were inversed when the cells were pretreated with anti-RAGE antibody.

**Conclusions:**

The current study suggest that AGEs can increase lipids accumulation in macrophages by regulating cholesterol uptake, esterification and efflux mainly through binding with RAGE, which provide a deep understanding of mechanisms how AGEs accelerating diabetic atherogenesis.

## Background

It is well established that people with diabetes mellitus have a greater risk of cardiovascular morbidity and mortality than their normal counterparts. More than 50 % of diabetes-related deaths are associated with macrovascular complications, especially atherosclerosis [1]. Multiple risk factors are associated with atherosclerosis, including hyperglycemia, hyperlipidemia, and dysregulation of the angiotensin system [2, 3]. Recent studies show that oxidative stress and advanced glycation also play an important role in the development of diabetic macrovascular disease [4, 5]. Advanced glycation end products (AGEs), a heterogeneous group of complex structures, form non-enzymatically when reducing sugars react with free amino groups on proteins, lipids, or nucleic acids. AGEs formation and accumulation, processes of normal aging driven by hyperglycemia, occur at an accelerated rate in diabetic patients and may participate in the pathogenesis of diabetic vascular disease [6]. The interaction between AGEs and its receptor, RAGE, contributes to the progression of atherosclerosis and accelerates oxidative stress [7–10]. RAGE, a member of the immunoglobulin superfamily of cell surface molecules, is a multi-ligand receptor on vascular cells that plays a key role in inflammatory processes [11]. Unlike other receptors that are downregulated by increased levels of their ligands, the RAGE-ligand interaction leads to positive feedback activation which further increases receptor expression [12]. There is increasing evidence that in diabetic patients, activation of the AGEs-RAGE pathway plays a central role in the cascade of events that result in accelerated atherosclerotic plaque formation, plaque erosion and fissuring [13, 14].

Atherosclerosis is a chronic disease characterized by the deposition of excessive cholesterol in the arterial intima. Macrophage foam cells play a critical role in the occurrence and development of atherosclerosis. Macrophage cholesterol homeostasis maintenance is the result of a balance among influx, endogenous synthesis, catabolism, and efflux [15]. Scavenger receptors (SRs), CD36 and SR class A (SR-A) are the principal receptors responsible for the binding and uptake of ox-LDL in macrophages [16]. ACAT1 and neutral cholesteryl ester hydrolase (nCEH) play a critical role in cholesterol esterification [17]. ATP-binding cassette (ABC) transporter A1(ABCA1), ABCG1 and SR-BI mediate cholesterol export from macrophages [18]. Expressions of ABCA1, ABCG1 and apoE are mediated through the nuclear receptors, liver X receptor (LXR) α and PPAR γ [19]. Besides that, HMG-CoA reductase (HMGCR), a rate-limiting enzyme for cholesterol synthesis, can also exerts its regulation effect on cholesterol synthesis at the transcriptional level [20]. It is related with the free cholesterol (FC) level of macrophage [21, 22]. When inflow and esterification of cholesterol increase and/or its outflow decrease, excessive esterified cholesterol (CE) will accumulate as lipid droplets in macrophages, thereby contributing to the formation of foam cells which initiate atherosclerosis.

Our previous study has found that AGEs-RAGE axis can increase the lipid droplets accumulation in the aortas of diabetic rats [23]. In that study, we observed obvious lipid droplets in the cytoplasm of smooth muscle cells and basal membrane of aortas when there were not any classical pathological changes of atherosclerosis can be seen by HE stainning in high-diet fed diabetic Goto Kakisaki (GK) rats. The expression of RAGE was also increased in aortas of GK rats. Recent experimental evidence also suggests that AGEs may promote foam cells formation [24–26]. However, the roles of AGEs in influencing the uptake, synthesis and efflux of cholesterol in macrophages and the underlying mechanisms are poorly understood. In the present study, we intended to investigate the effect of AGEs on lipid accumulation in macrophages stimulated with oxidized-LDL (oxLDL). Furthermore, we investigated the effects of AGEs-RAGE interaction on regulating cellular cholesterol efflux, influx, and esterification in THP-1 macrophages. This study will provide a new strategy for the treatment of diabetic atherosclerosis.

## Methods

### Preparation of model AGEs

BSA and D-glucose (Sigma, USA) were dissolved in phosphate-buffered saline PBS (pH 7.2–7.4), and the final concentrations of BSA and D-glucose was 5 g/L and 50 mmol/L, respectively. EDTA was added to a final concentration of 0.5 mmol/L to reduce oxidation. Penicillin (100 U/L) and streptomycin (100 μg/ml) were added to the reaction mixture to prevent bacterial contamination. The reaction mixture was filtered through 0.22 μm filter and then incubated in electrothermal incubation at 37 °C for 12 weeks. At the end of the incubation period, the reaction mixture was dialyzed against sterilized PBS (pH 7.2–7.4) to remove the unconjugated glucose; the glucose in the dialyzate was <0.03 mmol/L. The reaction mixture was measured in a fluorospectrophotometer with an excitation wave of 370 nm, and the maximum absorption peak was measured at 440 nm to verify that the mixture was AGE-BSA. Finally, the AGE-BSA was freeze-dried and stored at 4 °C.

### Cell culture and treatment

Human monocytic cell line THP-1 was purchased from Scientific Research Institute, Shanghai, China and cultured in RPMI 1640 medium containing 10 % FBS, 10 mM HEPES (Sigma, USA), and 1 % pen/strep solution at a density of 5 × 10^5^cells/ml in a 5 % CO2 incubator. The cells were seeded in six-well plates for 48 h in the presence of 100 ng/ml PMA, (Sigma, USA) which allowed them to differentiate into adherent macrophages. The culture medium was then changed into RPMI1640 medium containing 0.1 % FBS for 6 h of cell starvation. The macrophages were pretreated with BSA (600 μg/ml) as control group or with different concentration of AGEs (300 μg/ml and 600 μg/ml) for 2 h, then stimulated with oxLDL (100 μg/ml) (Jingke Chemistry, China) for 24 h at 4 °C in a 5 % CO2 incubator, and then collected for detection. To observe the effect of RAGE, one group of cells was treated with anti-RAGE antibody (10 μg/ml)(Abcam, USA) for 2 h before adding high concentration of AGEs (600 μg/ml).

### Oil red O staining

After the above mentioned treatment, the cells were collected, washed twice with ice-cold PBS, and then fixed with 10 % neutral-buffered formalin for 15 min. The cells were washed again with 60 % isopropyl alcohol for 1 min and then stained with oil red O (Sigma, USA) for 15 min. The oil red O was discarded, and excess dye was washed away with 60 % isopropyl alcohol. After washing with distilled water, the specimens of each group were placed under an inverted microscope to observe oil red staining. Cellular lipid accumulation was quantified by the optical density(OD) at 518 nm of Oil Red O extracted from stained cells using isopropanol. The results were normalized as OD value/number of cells.

### RNA isolation and quantitative RT-PCR

Total RNA was isolated using Trizol reagent (Invitrogen, CA). For reverse transcription, 2 μg of the total RNA was converted to first strand complementary DNA in 20 μl reactions using a reverse transcription kit (Thermo, USA). Quantitative real-time PCR analysis was performed (StepOne, Applied Biosystems) using SYBR Green (Takara, JAP). The thermal cycling program was 10 min at 95 °C for enzyme activation and 40 cycles of denaturation for 15 s at 95 °C, 5 min at 60 °C for annealing and extension. The comparative cycle threshold (CT) method was used to determine relative mRNA expression of genes as normalized by GAPDH housekeeping gene. Gene expression was determined by the 2^-ΔΔCt^ method. Primers used were as follows: 5’-GAAACTGAACACAGGCCGGA-3’ for RAGE-F, 5’- CACGGACTCGGTAGTTGGAC-3’ for RAGE - R; 5’ – TAAGATCAGGTGGG TTGGGC -3’ for SRA2-F; 5’ –CAGGTACAACACGGGAACCA -3’ for SRA2-R; 5’ –TCCTCTGGCAACAAACCACA-3’ for CD36-F; 5’ –AAGTCCTACACT GCAGTCCTC -3’ for CD36-R; 5’ –TGTCTGATGGCCGCTTTCT -3’ for ABCG1-F; 5’ –CACCTCATCCACCGAGACAC -3’ for ABCG1-R; 5’ –AGGGAGAG CACAGGCTTTGAC -3’ for ABCA1-F; 5’ –CCCCACTCACTCTCGCTCG -3’ for ABCA1-R; 5’ –TCTTATTGGTCGAAGGCTCGT -3’ for HMGCR-F; 5’ –TCTCACT AGAGGCCACCGAA -3’ for HMGCR-R; 5’ –AGCCAAGGTACAGGTAACGA -3’ for LXRα-F; 5’ –GATTACAACGGTGATGGCGG -3’ for - LXRαR; 5’ –TAG TCTACGCCTGTGGAGCC -3’ for ACAT-1-F; 5’ –TCTTATTTCCTG CACCAGCCTC -3’ for – ACAT-1- R; 5’ –GTAAACACGCTCCTCTAA -3’ for GAPDH-F; 5’ –TGACGGGATCTCGCTCCTGGAAGAT -3’ for GAPDH- R.

### Protein isolation and Western blot analysis

After treatment of cells as indicated above, cells were lysed with RIPA lysis buffer (50 mmol/L Tris pH 7.5, 150 mmol/L NaCl, 1 mmol/L EDTA, 1 % Triton X-100, 1 % sodium deoxycholate, 0.1 % SDS) containing protease inhibitor cocktail (Sigma, USA), and protein concentrations of lysates determined by the BCA assay kit (Beyotime, China). Proteins (20 μg of extracts) were electrophoresed by 12 % SDS-PAGE, and transferred to a nitrocellulose membrane. The membranes were blocked overnight at 4 °C in 5 % skim milk, and then incubated with primary antibodies against for RAGE, SRA2, CD36, ABCA1, ABCG1, LXRα, HMGCR and ACAT1 (1:1000), (Abcam, USA) 2 h at room temperature. The membranes were washed with TBS-T, and incubated with a horseradish peroxidase conjugated secondary anti-mouse or anti-rabbit IgG antibody (1:5000, Bioss, China) for 1 h at 37 °C. Afterwards blots were developed using ECL Plus. The signal intensities of specific bands were detected with a Clinx ChemiScope imaging-system (Qinxiang, China) and quantified using TotalLab TL100-Quick Start analysis software.

### Measurement of intracellular cholesterol and cholesterol esterification

After treatment of cells as indicated above, cells were washed twice with PBS, cellular lipids extracted with a mixture of hexane and isopropanol (3:2 v/v), and lipid extracts dried under a stream of nitrogen. Total cholesterol (TC) and FC concentrations were determined using enzymatic assay kit (Applygene Technologies Inc, China) in accordance with the manufacturer’s instructions. The concentration of CE was determined by subtraction of the concentration of free cholesterol from that of total cholesterol. Cholesterol concentrations were related to cellular protein as determined by the BCA protein assay kit (Beyotime, China). Cellular protein was obtained by lysis of cells in 0.2 mol/L NaOH after extraction of cellular lipids. Results are expressed as lipids/mg cellular protein.

### Cholesterol uptake assay

The treated macrophages were incubated with 1,1’-dioctadecyl- 3,3,3’,3’ -tetramethylin docarbocyaninet (Dil)-oxLDL (40 μg/ml) (Jingke Chemistry, China) instead of oxLDL for 24 h. After the medium was removed, cells were washed twice with PBS. The specimens of each group were placed under an inverted fluorescence microscope (OLYMPUS, CKX41, JAP). Fluorescence intensity was expressed as Mean fluorescence intensity (MFI)/number of cells, and MFI was caculated by the cumulative fluorescence density/area analysed by Image-Pro Plus 6.0. Fluorescent intensity was quantified from at least 3 random fields (1024 × 1024 pixels) per slide, from 3 slides per experimental condition and graphed.

### Cholesterol efflux assay

The treated macrophages were plated in 12-well plates and loaded with 40 μg/ml Dil-oxLDL for 48 h at room temperature. Then the medium was removed and cells were washed twice with PBS. The culture medium was then changed into RPMI1640 medium containing 0.1 % BSA supplemented with apoAI (10 μg/ml, Novoprotein, China) or HDL (100 μg/ml, Jingke Chemistry, China) at 37 °C for 24 h. At the end of this incubation period, the supernatant was collected and centrifuged at 12,000 rpm for 10 min to remove debris. Cells were lysed with 0.5 mL of 0.1 N NaOH. The fluorescence in both the supernatant and cellular lipid was measured on a fluorescence microplate (Thermo Labsystems, USA). Fluorescence intensity was determined and expressed as a percentage of the total cell Dil-oxLDL content (effluxed Dil-oxLDL plus cell-associated Dil-oxLDL).

### Statistical analysis

Data are expressed as mean ± SD. Results were analyzed by oneway ANOVA followed by Fisher's LSD procedure using SPSS 21.0. *P* < 0.05 was considered statistically significant. All experiments were performed at least three times.

## Results

### AGEs-induced up-regulation of RAGE expression in THP-1 macrophages

**Fig. 1:**
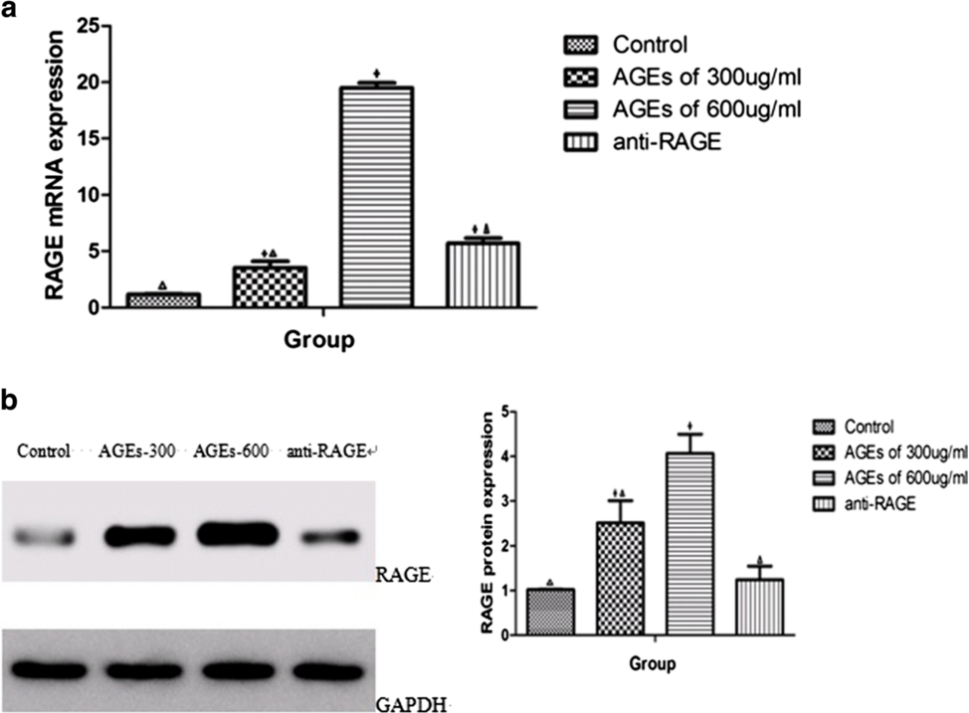
Effect of AGEs on RAGE expression in macrophages. **a** The mRNA expression of RAGE was determined using quantitative real-time PCR; **b** Protein expression of RAGE in each group was analyzed by Western blot. GAPDH demonstrated equal loading. The right panel shows the average densitometric analysis of three independent experiments. Data represent mean ± SD, from three independent experiments, each performed in triplicate. *P < 0.05 vs. control group. ^Δ^ *P* < 0.05 vs. 600 μg/ml AGEs group

After incubation with AGEs at different concentrations (300 μg/ml and 600 μg/ml), RAGE expression was substantially up-regulated both in mRNA (Fig. 1a) and protein (Fig. 1b) compared with control group in a concentration-dependent manner. There were 3.0-fold increase in mRNA (3.53 ± 0.57 *vs* 1.15 ± 0.07, *P* = 0.049) and 2.5-fold increase in proteins (2.52 ± 0.49 *vs* 1.02 ± 0.01, *P* < 0.001) compared with control group when the concentration of AGEs was 300 μg/ml. As the concentration of AGEs reaching at 600 μg/ml, increasement of RAGE expression was 17.0 times in mRNA (19.5 ± 0.43 *vs* 1.15 ± 0.07, *P* < 0.001) and 4.0 times in proteins (4.07 ± 0.43 *vs* 1.02 ± 0.01, *P* < 0.001) compared with control group. After pretreating with anti-RAGE antibody, RAGE expression was suppressed both in mRNA (5.70 ± 0.45 *vs* 19.5 ± 0.43, *P* < 0.001) (Fig. 1a) and protein (1.24 ± 0.31 *vs* 4.07 ± 0.43, *P* < 0.001) (Fig. 1b) compared with 600 μg/ml AGEs group.

### AGEs increase the oxLDL-induced lipid accumulation in THP-1 macrophages

**Fig. 2:**
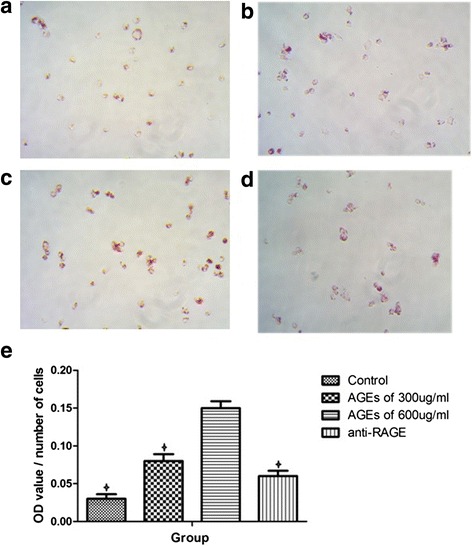
AGEs increase lipid accumulation in macrophages in a concentration-dependent manner. **a**–**d** Representative images of oil red staining. **a** control group; **b** AGEs of 300 μg/ml group; **c** AGEs of 600 μg/ml group; **d** AGEs of 600 μg/ml plus anti-RAGE antibody group. The magnification of each panel is 200×. **e** Quantification of Oil Red O stained cells as measured by the OD value at 518 nm. The results were normalized as OD value/number of cells. Data represent mean ± SD. from three independent experiments, each performed in triplicate. *P < 0.05 vs. 600 μg/ml AGEs group. One of three representative experiments is shown

Intracellular lipid droplets were observed through oil red staining. As shown in Fig. 2a–d, AGEs-stimulated macrophages showed much more red-stained lipid droplets than the control group, especially in the group of 600 μg/ml AGEs. However, this trend was inhibited by anti-RAGE antibody pretreatment. The optical density (OD) value of each group which was normalized by the number of cells showed that there was significant change in 600 ug/ml AGEs group compared with control group and anti-RAGE antibody pretreated group (Fig. 2 e).

### AGEs increase lipid uptake in THP-1 macrophages by up-regulating CD36, SRA2 expression mediated by RAGE

**Fig. 3:**
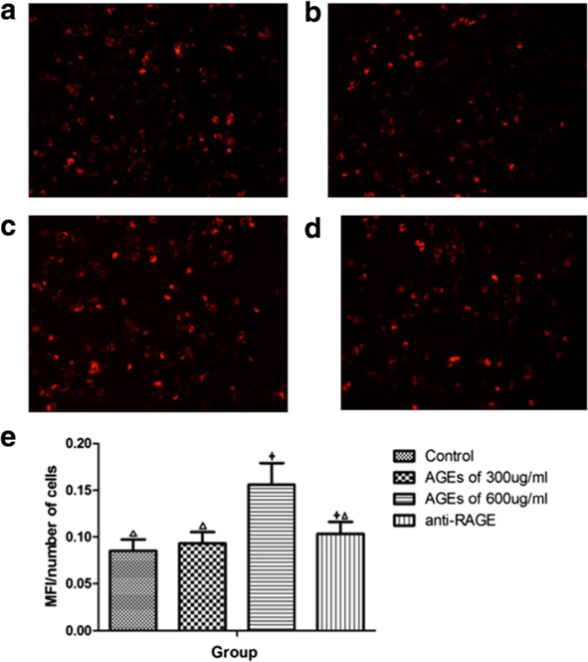
AGEs increase oxLDL uptake in macrophages. **a**–**d** Cells were analyzed on a OLYMPUS inverted fluorescence microscope system and presented as photomicrographs. **a** control group; **b** 300 μg/ml AGEs group; **c** 600 μg/ml AGEs group; **d** 600 μg/ml AGEs plus anti-RAGE antibody group. **e** Quantification of fluorescent intensity of cells as MFI/number of cells. Data represent mean ± SD, from three independent experiments. Fluorescent intensity was quantified from at least 3 random fields (1024 × 1024 pixels) per slide, from 3 slides per experimental condition and graphed. * *P* < 0.05 vs. control group. ^Δ^ *P* < 0.05 vs. 600 μg/ml AGEs group. One of three representative experiments is shown

To investigate the effects of AGEs on cholesterol uptake in macrophages, we examined cholesterol uptake using a Dil-oxLDL binding assay in response to the treatments with AGEs at the concentrations of 300 μg/ml and 600 μg/ml in THP-1 macrophages. As shown in Fig. 3a–c) and (e), AGEs at a concentration of 300 μg/ml did not significantly alter Dil-oxLDL binding to THP-1 macrophages (0.093 ± 0.012 *vs* 0.085 ± 0.012, *P* = 0.335), while 600 μg/ml AGEs significantly increased oxLDL internalization in THP-1 macrophages (0.156 ± 0.023 *vs* 0.085 ± 0.012, *P* < 0.001). Pretreatment with anti-RAGE antibody before adding AGEs in concentration of 600 μg/ml reduced Dil-oxLDL binding to THP-1 macrophages compared with only AGEs (0.103 ± 0.013 *vs* 0.156 ± 0.023, *P* < 0.001) (Fig. 3d, e).

**Fig. 4:**
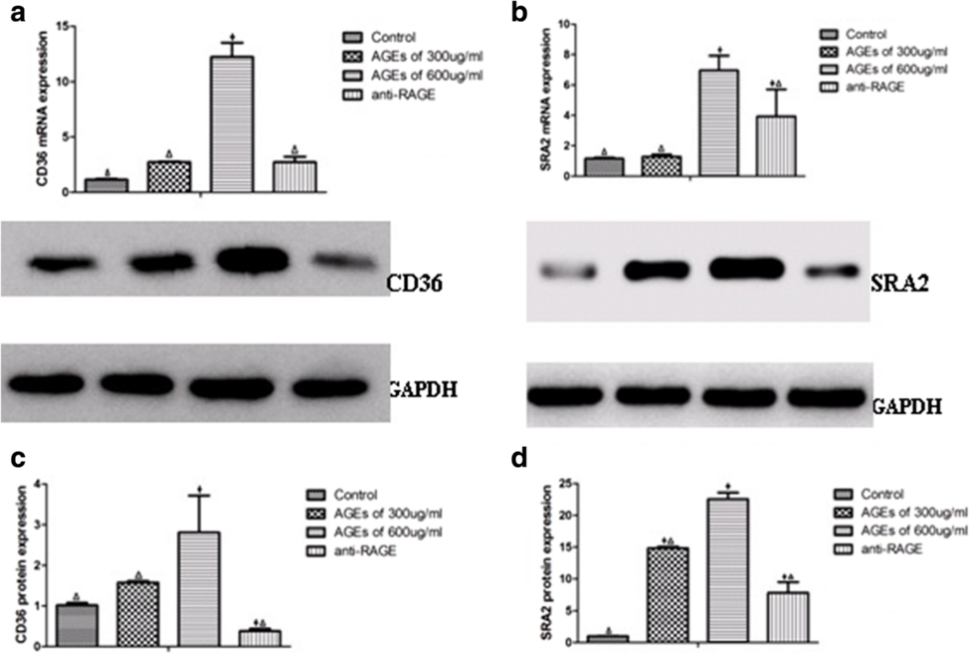
AGEs upregulate CD36, SRA2 expression in macrophages. The expression of CD36, SRA2 mRNA (**a**, **b**) and protein (**c**, **d**) were determined using real-time quantitative PCR and western blotting assays, respectively. All the results were expressed as mean ± SD, from three independent experiments, each performed in triplicate. *P < 0.05 vs. control group. ^Δ^ *P* < 0.05 vs. 600 μg/ml AGEs group

Macrophages phagocytose oxLDL through scavenger receptors, including CD36, and scavenger receptor A, to form foam cells. To elucidate the molecular mechanism of AGEs on the accumulation of oxLDL in macrophages, we detected the expression of scavenger receptors, CD36 and SRA2. As depicted in Fig. 4, AGEs in concentration of 600 μg/ml can dramatically increase CD36 and SRA2 mRNA and protein levels (*P* <0.05); whereas, those changes can’t be observed in cells incubated with 300 μg/ml AGEs. The application of antibody of RAGE significantly suppressed the expression of CD36 and SRA2 both in mRNA and protein compared with only AGEs of 600 μg/ml (*P* <0.05) (Fig. 4).

### AGEs reduce ABCG1-dependent cholesterol efflux to HDL through interaction with RAGE

**Fig. 5:**
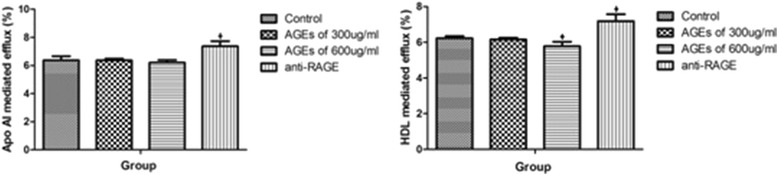
AGEs decreased cholesterol efflux to HDL but not to apoAI in macrophages. Fluorescence intensity was determined and expressed as a percentage of the total cell Dil-oxLDL content. Data represent mean ± SD, from three independent experiments, each performed in triplicate. *P < 0.05 vs. control group

We next investigated the effect of AGEs on cholesterol efflux. By using Dil-oxLDL, we found that AGEs in concentration of 600 μg/ml elicited a significant reduction in cholesterol efflux mediated by HDL but not apoAI, expressed as the percentage of total cell cholesterol released into the medium (*P* < 0.05). Treatment with antibody of RAGE significantly increased cholesterol efflux (*P* < 0.05). (Fig. 5)

**Fig. 6:**
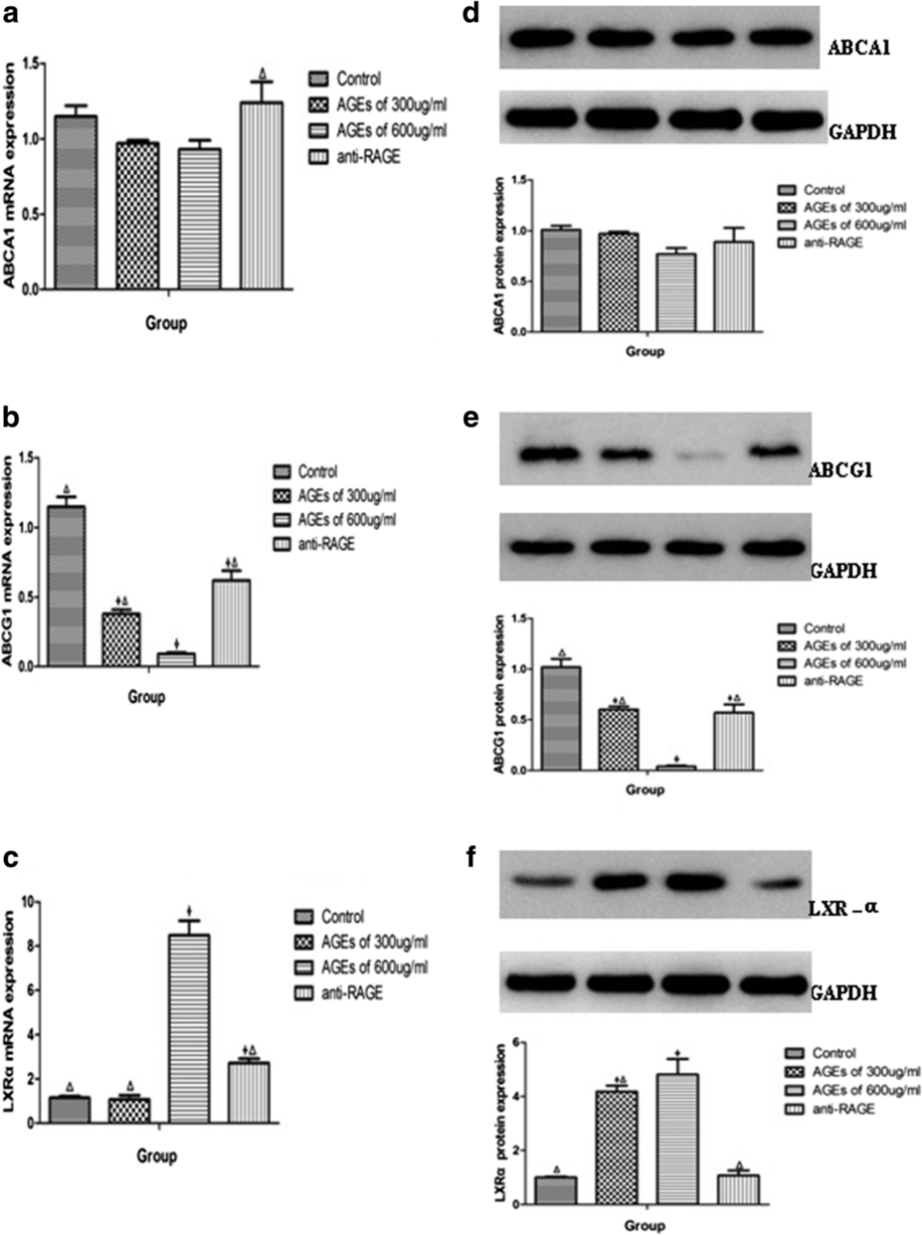
AGEs downregulate ABCG1 expression in macrophages. The expression of ABCA1, ABCG1, LXR-α mRNA (**a**–**c**) and protein (**d**–**f**) were determined using real-time quantitative PCR and western blotting assays, respectively. All the results are expressed as mean ± SD, from three independent experiments, each performed in triplicate. *P < 0.05 vs. control group. ^Δ^ *P* < 0.05 vs. 600 μg/ml of AGEs group

Then we examined the expression of both ABCA1 and ABCG1 in macrophages. AGEs significantly reduced the mRNA expression of ABCG1 but not ABCA1 in concentration- depended manner (300 μg/ml (0.38 ± 0.03 *vs* 1.15 ± 0.07,*P* < 0.001) and 600 μg/ml of AGEs (0.09 ± 0.01 vs 1.15 ± 0.07,*P* < 0.001) (Fig. 6a, b). Pretreating THP-1 macrophages with an anti-RAGE antibody significantly suppressed this effect (Fig. 6b). The expression of ABCG1 in protein was in accordance with that in mRNA (Fig. 6e).

Then we examined the potential involvement of transcription factors LXR-α, known regulators of ABCA1 and ABCG1 expression in macrophages. We found that expression of LXR-α was up-regulated both in mRNA and in protein in incubation with AGEs, and the pretreatment with anti-RAGE antibody suppressed the effect (Fig. 6c, f).

### AGEs increase cholesteryl ester formation by targeting ACAT1 and HMGCR through regulating RAGE expression

**Fig. 7:**
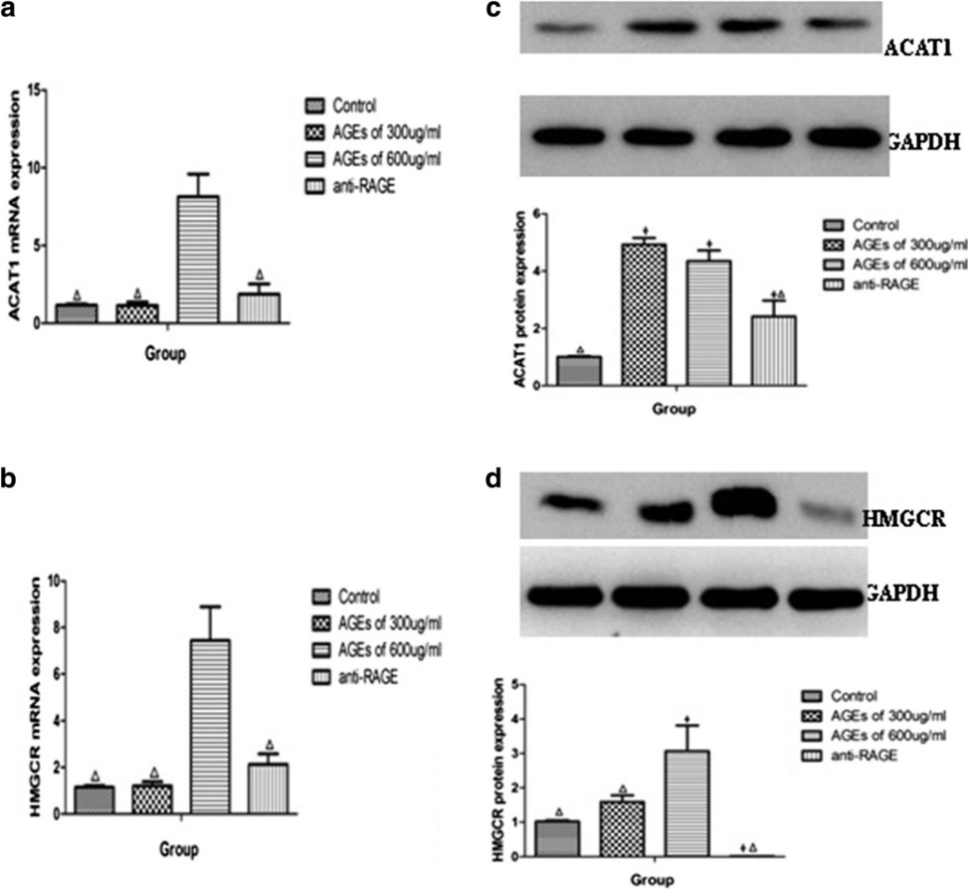
AGEs upregulate ACAT-1, HMGCR expression in macrophages. The expression of ACAT-1, HMGCR mRNA (**a**, **b**) and protein (**c**, **d**) were determined using real-time quantitative PCR and western blotting assays, respectively. All the results are expressed as mean ± SD, from three independent experiments, each performed in triplicate. *P < 0.05 vs. control group. ^Δ^ *P* < 0.05 vs. 600 μg/ml AGEs group

We measured the levels of TC and FC by enzymatic assay kit and calculated the levels of CE. The data (Table 1) showed that 600 μg/ml of AGEs treatment increased CE levels compared with the control group (*P* < 0.001). However, pretreatment with anti-RAGE antibody inhibited AGEs-induced foam cell formation (*P* < 0.05). We further investigated the expression of HMGCR and ACAT-1, which were related with the endogenous synthesis and esterification of FC in THP-1 macrophages. AGEs significantly reduced ACAT1 mRNA expression in a high concentration of 600 μg/ml (8.15 ± 1.45 *vs* 1.15 ± 0.07,*P* < 0.001) but reduced ACAT1 protein levels even in a low concentration of 300 μg/ml (4.93 ± 0.23 *vs* 1.02 ± 0.06,*P* < 0.001) (Fig. 7a, c). The expressions of HMGCR in mRNA and protein were increased in concentration of 600 μg/ml of AGEs (*P* < 0.05) (Fig. 7b, d). Pretreatment with anti-RAGE antibody showed the opposite effect, significantly decreasing ACAT1 and HMGCR levels (Fig. 7a–d).

**Table 1:** Effects of AGEs on cholesterol content in macrophages. (ng/μg protein)

	Control	300 μg/ml of AGEs	600 μg/ml of AGEs	600 μg/ml of AGEs + Anti-RAGE antibody
TC	197.13 ± 22.1**	234.43 ± 11.23**	652.61 ± 11.76*	407.78 ± 33.98*^,^ **
FC	163.21 ± 7.42**	201.83 ± 12.44**	458.70 ± 29.14*	284.20 ± 33.57*^,^ **
CE	33.92 ± 29.49**	32.6 ± 20.02**	193.91 ± 31.39*	123.58 ± 65.19*^,^ **

Data are expressed as mean ± S.D. *P < 0.05 vs. control group, **P < 0.05 vs. 600 μg/ml of AGEs group

## Discussion

The maintain of macrophage cholesterol homeostasis is of great importance in the prevention of atherosclerosis. Dysregulation of the balance of cholesterol influx, endogenous synthesis, esterification/hydrolysis, and cholesterol efflux leads to excessive accumulation of cholesterol in macrophages and their transformation into foam cells and death [15]. In the present study, we elucidated the underlying mechanisms of AGEs-RAGE regulated cellular influx, intracellular esterification/hydrolysis and efflux of cholesterol. Our results provide strong evidence that AGEs-RAGE interaction may regulate the processes of cholesterol homeostasis from influx to efflux by increasing the expression of SRA2, CD36, ACAT1, HMGCR and decreasing expression of ABCG1 in macrophages.

There is increasing evidence that AGEs and their interaction with RAGE play a pivotal role in atherosclerosis, in particular in the setting of diabetes. AGEs binding to RAGE activates various signalling pathways, including NADPH oxidases, mitogen-activated protein kinases (MAPKs), p21^ras^, ERK p38 and protein kinase C (PKC), and finally leads to sustained cellular dysfunction driven by long-term activation of the nuclear factor-kB (NF-kB) [27, 28]. The importance of AGEs as downstream mediators of hyperglycaemia in diabetes has been amply demonstrated by animal studies using inhibitors of advanced glycation to retard the development of vascular disease without directly influencing plasma glucose levels [29, 30]. Furthermore, dietary excess of AGEs has been shown to accelerate atherosclerosis without affecting glycaemic control [31]. Studies in vivo showed that administration of soluble RAGE (sRAGE), a truncated form of RAGE acting as a decoy for AGE, completely suppressed diabetic atherosclerosis in glycemia- and lipid-independent manners [13]. In diabetes-associated atherosclerosis models, RAGE overexpression in transgenic mice was associated with increased vascular injury, while RAGE deletion conferred partial vascular protection [32].

Despite the important contribution of AGE to the accelerated atherosclerosis in diabetes, the specific molecular mechanisms in response to AGE within macrophages (a central player in atherogenesis) remain unclear. In this study, we discovered that exposure of THP-1 macrophages to AGEs was concentration-dependently associated with lipid accumulation observed by oil O stain and intracellular CE/TC determination. At the same time, the expression of RAGE elevated in parallel with the increase of lipid accumulation. RAGE specific antibody led to reversions in lipid contents in macrophages, suggesting that AGE-elicited atherogenic effects in macrophages were, at least partly, RAGE-dependent. These observations suggest that excessive formation of AGEs could render diabetic patients under high risk of developing atherosclerosis.

The Dil-oxLDL binding assay in our study showed that AGEs increased the binding of Dil-oxLDL to macrophages, indicating that AGEs promote cholesterol uptake. Cholesterol uptake is a pathway by which extracellular modified LDL are ingested by macrophages via receptors-mediated phagocytosis and pinocytosis. SRs such as SR-A and CD36 have been implicated in this process. In vitro studies have shown that CD36 and SR-A account for 75–90 % of ox-LDL internalization by macrophages, whereas other SRs cannot compensate for their absence [33]. In present study we found AGEs can upregulated SRA2 and CD36 mRNA and protein levels, indicating that AGEs may promote cholesterol uptake by increasing SRA2 and CD36 expression. It has been reported that AGEs only without interacting with RAGE can up-regulate the expression of SRA2 and CD36 [34, 35]. However, It was not the same in our study. After blockade of RAGE by specific antibody, we found that SRA2 and CD36 expression decreased significantly accompanied with the reduction of cholesterol uptake, suggesting that AGEs increase cholesterol uptake of macrophages mainly through binding with RAGE. A unique feature of CD36 is that expression of its gene could be regulated by ligands via PPAR-γ dependent signaling pathway, and SRA-2 is mainly regulated by PPAR-γ and NF-kB signaling pathway [33]. Activation of RAGE with AGEs leads to the production of reactive oxygen (ROS) and nitrogen species (RNS) by a variety of mechanisms, which may activates the PPAR-γ and NF-kB signaling pathway [24] [36]. It may be the molecular mechanism for upregulation of CD36 and SRA2 by AGEs-RAGE interaction.

The results of cholesterol efflux assay in our study showed that AGEs can reduce cholesterol efflux through HDL but not apo AI in macrophages. When blocking AGEs-RAGE interaction by anti-RAGE antibody, the decrease of cholesterol efflux would recover. It is confirmed that AGEs have deleterious effects on cholesterol efflux in macrophages through binding with RAGE. Then we determined the transporters associated with cholesterol efflux and found that AGEs decreased ABCG1 mRNA and protein levels in macrophages in a RAGE-dependent manner.

Reverse cholesterol transport (RCT) is the primary pathway for the removal of excess cholesterol and involves lipid transporters such as ABCA1 and ABCG1 that mediate the transfer of cholesterol from peripheral cells to selected extracellular acceptors [37, 38]. The full ABC transporter ABCA1 appears most effective at mediating cholesterol efflux to apoAI as acceptor [39, 40]. The “half transporter” ABCG1 also facilitates cholesterol efflux from macrophages, preferring HDL as acceptor [41, 42]. LXR can control the expression of both ABCA1 and ABCG1 [43], and several recent reports suggest that both transporters share similar transcriptional control mechanisms [44, 45]. The effects of AGEs-RAGE axis on ABC transporters reported in previous studies are contradictory. The results of Passarelli M et al. [46] have shown that AGEs can impair cholesterol efflux from cultured human fibroblasts and murine macrophages through suppressing ABCA1 expression [46]. However, Isoda K et al. [47] reported that AGEs reduced macrophage cholesterol efflux to HDL through decreasing ABCG1 expression in a LXR-α independent way [47]. Recent study from Daffu G et al. [48] have demonstrated that RAGE suppressed macrophage cholesterol efflux in diabetic animal models and several different cell lines by moderately upregulating ABCA1 expression and significantly upregulating ABCG1 expression in a LXRα-independent way [48]. Our results were more like Daffu G’s that ABCA1 expression was slightly changed without statistical significance after treatment, while ABCG1 expression was dramatically changed with obviously statistical significance. LXR-α expression was contrary to both ABCA1 and ABCG1. So we report here that AGEs mainly reduces the expression of ABCG1 but not ABCA1 in a LXRα-independent manner in THP-1 macrophages, supporting the notion that ABCG1 may be especially important in diabetic atherosclerosis and providing a novel mechanistic insight into the relationship between HDL and atherosclerosis risk in diabetic patients. Our results also provide evidence that there are other signaling pathway to regulate expression of ABCG1 except LXR-α. Peroxisome proliferator response elements (PPRE) in the promoter regions of target genes is recognized as a important regulator which can binging with PPAR-γ to activate transcription [49]. It has been demonstrated that RAGE ligands suppressed ABCG1 and ABCA1 promoter luciferase activity and transcription of ABCG1 and ABCA1 through PPRE but not LXR elements [48]. So it can be speculated from our results that AGEs-RAGE axis may regulate ABCG1 expression mainly through PPRE binding with PPAR-γ, while ABCA1 expression may be mainly controlled by classical PPAR-γ/LXR-α signaling pathway which is less affected by AGEs-RAGE axis. Besides PPRE, previous findings suggest a hitherto unsuspected degree of complexity in the regulation of the ABCG1 gene. The human ABCG1 gene spans 97 kb comprising 20 exons, potentially giving rise to multiple transcripts, and further contains two promoters with binding sites for multiple transcription factors, including NF-kB and sterol regulatory element-binding protein (SREBP) [50]. Both SREBP and NF-kB have been shown to be involved in the coactivation of transcription of genes involved in cholesterol metabolism [51]. Such structural complexity raise speculation about the potential mechanism that AGEs-RAGE axis interfere with the expression of ABCG1 through NF-kB and SREBP signaling pathway. Further studies will be required to elucidate the details of AGEs-RAGE-NF-kB/ SREBP-ABCG1 regulating pathway.

In the process of foam cell formation, ACAT1 re-esterifies excess FC to promote the biosynthesis of CE that is stored in lipid droplets [52]. In this study, we found that AGEs increased cellular CE levels. Furthermore, we demonstrated that high concentration of AGEs targeted expression of ACAT1, regulated the mRNA and protein levels of ACAT1, and increased CE formation in macrophage-derived foam cells. In contrast, low concentration of AGEs increased the protein expression of ACAT1 but had no effect on its mRNA expression, which can’t be explained by present study. However, it should be noted that ACAT1 protein increased similarly after treatment with 300 and 600 μg/ml AGEs, but the CE levels didn’t increase over control at 300 μg/ml AGEs. The possible reseason for that may be the difference of internalization of lipoproteins by macrophages between 300 and 600 μg/ml AGEs group. Besides, we didn’t detect the expression of nCEH which hydrolyzes CE to cholesterol for efflux out of the cells. The changes of nCEH caused by different concentration of AGEs can also affect the levels of CE. In addition to uptake of extracellular lipoprotein, another main sources of intracellular FC are endogenous synthesis, which is regulated by HMGCR, a rate-limiting enzyme in the pathway for cholesterol synthesis [53]. Our study have demonstrated that AGEs can upregulate expression of HMGCR which can increase intracellular cholesterol and promote CE formation. Both ACAT1 expression and HMGCR expression decreased when using anti-RAGE antibody to pretreat, which indicated that the role of AGEs in upregulating ACAT1 and HMGCR needed to bind with RAGE. SREBP2 is probably the key factor connecting AGEs/RAGE with ACAT1/HMGCR [54].

## Conclusions

In conclusion, the present study shows that AGEs increase lipids accumulation in macrophages probably by upregulating RAGE expression. The whole process of foam cell formation including cholesterol influx, synthesis and efflux in macrophage can be affected by AGEs-RAGE interaction. Our study provide a deep understanding how AGEs can accelerate diabetic atherogenesis and suggest that drugs for anti-AGEs, downregulating expression of RAGE or blocking AGEs binding with RAGE may be useful adjunctive therapeutic agents in the management of diabetic atherosclerosis especially in the early stage to prevent foam cell formation. According to the results that most of the changes induced by AGEs are concentration dependent, our study emphasize the importance of good blood glucose control which can reduce AGEs levels both in blood serum and in arterial wall in prevention of diabetic atherosclerosis. It is also indicated that HDL may have special meaning for diabetic patients compare with non-diabetic people. Further studies need to be done to understand the detailed signaling pathways whereby AGEs-RAGE breaks the homeostasis of cholesterol metabolism in macrophages.

## Abbreviations

ABCA1: ATP-binding cassette transporter A1
ABCG1: ATP-binding cassette transporter G1
AGEs: Advanced glycation end products
CE: Esterified cholesterol
Dil: 1,1’-dioctadecyl- 3,3,3’,3’ -tetramethylin docarbocyaninet
FC: Free cholesterol
HMGCR: HMG-CoA reductase
LXR: Liver X receptor
MAPKs: mitogen-activated protein kinases
MFI: Mean fluorescence intensity
nCEH: Cholesteryl ester hydrolase
NF-kB: Nuclear factor-kB
OD: Optical density
oxLDL: oxidized-LDL
PKC: Protein kinase C
PPRE: Peroxisome proliferator response elements
RAGE: Receptor of advanced glycation end products
SR: Scavenger receptors
SREBP: Sterol regulatory element-binding protein
TC: Total cholesterol
